# Missed Ureteral Stents and Related Problems

**DOI:** 10.5812/numonthly.9420

**Published:** 2013-03-30

**Authors:** Siavash Falahatkar

**Affiliations:** 1 Urology Research Center, Guilan University of Medical Sciences, Rasht, IR Iran

**Keywords:** Ureter, Stents

Dear Editor,

The author has reported the management of a large group of forgotten ureteral stents. The paper has a capacity to remind the complications of missed stents. The paper entitled “Combined Percutaneous and Transurethral Lithotripsy for Forgotten Ureteral Stents with Giant Encrustation” has been written apparently (1). The author has mentioned by endourological procedures, the success would be achieved in the majority of patients but precluding the mistakes is advised.

Nowadays the usage of ureteral stent in the diagnosis and treatment of numerous urologic diseases is mandatory; as a result, its complications would be increased. Ureteral stent is used frequently in urologic procedures especially following a complicated TUL. The evaluation and solution of these concomitant complications are requisite. Some prudent manners should be considered during the procedure and after that the surgeon should be careful not to use force while inserting the ureteral stent. The patient should imbibe high fluid to prevent UTI and encrustation. It is recommended to prescribe prophylactic antibiotic which can prevent UTI. Active or symptomatic infection must be treated forthwith, for avoiding septicemia and encrustation of the stent. Forgotten ureteral stents is a dilemma. Migration, perforation of urinary tract, penetration to adjacent organs, malposition, UTI, ureteral erosion or fistulization, encrustation, stone formation, and fractured stent are the other major complications (2, 3).

Stony stent or complete stent encrustation is a serious complication of prolonged missed ureteral stent and it often needs to be managed by endoscopic techniques. However, open surgery is the final option. Occasionally, forgotten ureteral stents can create a medicolegal problem. In one study Diamond-like carbon coating surface effectively decreased encrustation tendencies of the stent (4). Because of the serious complications of Stony stent or complete stent encrustation, it is important to manage in a discreet manner.

According to the indication and physician experience, the stent would be placed for a short or long period of time. Many manufacturers usually recommend the stent exchanging at a 3- to 6 month intervals. It has shown the prevalence of complications would be increased as the indwelling time rises (5).

There are several approaches to managing a forgotten ureteral stent. When the surgeon is faced with encrusted or stony stent, it is requisite to consider several scenarios to cope with the problem, for example cystolithotripsy, ESWL, TUL, PCNL, laparoscopic or open surgery, etc. Although ESWL has become more attractive, the debate of its efficacy has not reached the pinnacle. When the stone is small and only in the kidney, the result of ESWL can be more effective. It seems the duration of stent can affect the results of ESWL, the more duration of stent placing, the more hardness of encrustation.

The surgeon will be able to treat a lot of patients by endourological procedures. Bladder is one of the common places for an encrusted or stony stent. Most of the time, endourological procedures are able to solve problems but open surgery may be occasionally needed. Often cystolithotripsy is successful and the remained stent can be removed easily. Occasionally, the stent will be removed with no difficulty after that the surgeon will discern the encrustation. TUL is a useful technique to fragment the encrusted or stony stent.

In the presence of large formed stones in the bladder and kidney, a combined procedure such as TUL and PCNL might be imperative. PCNL can be performed with TUL at the same time, if the surgeon has the experience of csPCNL (6). There are a few indications for open surgery and laparoscopic surgery to cope with the stony stents.
